# Spectroscopic Discrimination of Sorghum Silica Phytoliths

**DOI:** 10.3389/fpls.2019.01571

**Published:** 2019-12-11

**Authors:** Victor M. R. Zancajo, Sabrina Diehn, Nurit Filiba, Gil Goobes, Janina Kneipp, Rivka Elbaum

**Affiliations:** ^1^School of Analytical Sciences Adlershof (SALSA), Humboldt-Universität zu Berlin, Berlin, Germany; ^2^Chemistry Department, Humboldt-Universität zu Berlin, Berlin, Germany; ^3^BAM Federal Institute for Materials Research and Testing, Berlin, Germany; ^4^Department of Chemistry, Bar Ilan University, Ramat Gan, Israel; ^5^R. H. Smith Institute of Plant Sciences and Genetics in Agriculture, The Hebrew University of Jerusalem, Rehovot, Israel

**Keywords:** phytoliths, biosilicification, Raman, sorghum, solid state NMR, synchrotron FTIR

## Abstract

Grasses accumulate silicon in the form of silicic acid, which is precipitated as amorphous silica in microscopic particles termed phytoliths. These particles comprise a variety of morphologies according to the cell type in which the silica was deposited. Despite the evident morphological differences, phytolith chemistry has mostly been analysed in bulk samples, neglecting differences between the varied types formed in the same species. In this work, we extracted leaf phytoliths from mature plants of *Sorghum bicolor* (L.) Moench. Using solid state NMR and thermogravimetric analysis, we show that the extraction methods alter greatly the silica molecular structure, its condensation degree and the trapped organic matter. Measurements of individual phytoliths by Raman and synchrotron FTIR microspectroscopies in combination with multivariate analysis separated bilobate silica cells from prickles and long cells, based on the silica molecular structures and the fraction and composition of occluded organic matter. The variations in structure and composition of sorghum phytoliths suggest that the biological pathways leading to silica deposition vary between these cell types.

## Introduction

Grasses are silicon accumulators, concentrating silicic acid (herein Si) from the soil solution through the activity of Si transporters (Ma et al., 2006; Ma et al., 2007; Sakurai et al., 2015). Si moves with the water transpiration stream and deposits as hydrated amorphous silica (SiO_2_·nH_2_O) impregnating cell walls and filling cell lumens and intercellular spaces (Prychid et al., 2003). These microparticles are termed phytoliths. We can find phytoliths in root endodermis, leaf epidermis, inflorescence bracts, preferentially in highly transpiring organs (Jones et al., 1963). Phytoliths studies are relevant to geology and archaeology. This is because, similarly to pollen grains, under ambient conditions they are the more stable than other plant parts (Kelly et al., 1991; Shahack-Gross et al., 1996; Albert et al., 1999; Elbaum et al., 2003; Piperno et al., 2009; Ball et al., 2016). Organic molecules are trapped within phytoliths (Perry, 1985; Harrison, 1996; Elbaum et al., 2009; Parr and Sullivan, 2010; Gallagher et al., 2015; Asscher et al., 2017) and possibly reflect the chemical environment in which the silica formed (Perry and Keeling-Tucker, 2000). These organic entities can be studied by nuclear magnetic resonance (NMR) (Ravera et al., 2016). A seminal study of the hairs in the grass *Phalaris canariensis* demonstrates that plant silica has a significant fraction of surface silanol groups (Mann et al., 1983; Perry and Mann, 1989).

In order to study phytoliths, the plant tissue around them is digested, many times by harsh chemistry, high temperature, or mild chemistry during very long time periods (archaeologic or geologic). These processes change the physical and chemical properties of phytoliths. These changes were monitored in phytolith assemblies (Jones and Milne, 1963; Cabanes et al., 2011; Watling et al., 2011; Cabanes and Shahack-Gross, 2015). Individual phytoliths were also characterized (Perry et al., 1984a; Perry et al., 1984b; Elbaum et al., 2003; Watling et al., 2011; Alexandre et al., 2015; Gallagher et al., 2015), and variation in the mineral structure was identified within one phytolith type (Perry et al., 1990). However, different phytolith morphotypes were not compared, and we do not know whether a specific morphotype has a unique chemical signature, which is different from other morphotypes.

Raman and fourier transformed infrared (FTIR) microspectroscopy enable the probing of individual phytoliths and assessing their mineral structure and occluded organic matter. In these vibrational microspectroscopy methods, information on chemical bonds and thereby structure and composition of a sample is obtained. By combining a microscope with FTIR or Raman spectrometer, the spectra are collected at a micrometre resolution. FTIR absorption spectroscopy gives fingerprint-like information that has been widely used to study cell wall constituents like proteins, aromatic phenols, cellulose, and to characterize biologically produced silica (also referred to as biogenic silica or biosilica, (e.g. Fröhlich, 1989; Kačuráková et al., 2000; Gendron-Badou et al., 2003; Kerr et al., 2013). Raman spectroscopy complements the information from FTIR spectroscopy and was used to analyse cell wall polymers, silica, phenolics, and lipids in varied plant tissues (e.g., Sapei et al., 2007; Chylińska et al., 2014; Prats Mateu et al., 2016). Spectral information is often encoded in very minute features. Principal component analysis (PCA) transforms the spectral dataset into a variance weighted vector-space, and provides us with a highly sensitive analysis for subtle spectral variations.

In this work, we extracted silica phytoliths from sorghum leaves, using two wet digestion methods, and compared the extracts using bulk and individual phytolith analyses. We used vibrational microspectroscopy, both Raman and FTIR, to characterize individual phytoliths and evaluate the differences between phytolith morphotypes. Our results indicate a significant influence of the extraction method on the structure and composition of phytoliths silica and occluded organic matter. Nonetheless, we could show that specific phytolith morphotypes contain characteristic organic molecules.

## Material and Methods

### Controlled Plant Growing Conditions

Seeds of *Sorghum bicolor* (L.) Moench (line BTx623) were sown in 1-L pots in universal potting soil (Bental 11, Tuff Merom Golan), and grown in a greenhouse at The Robert H. Smith Institute of Plant Sciences and Genetics greenhouse in Rehovot, Israel during September 20 2016 to January 1, 2017 under natural light and temperature of the Israeli autumn (21°C –33°C). The plants were irrigated automatically twice a day by water supplemented with N-P-K fertilizer (nitrogen (N), phosphorus (P_2_O_5_), and potassium (K_2_O)) at respective % weight ratio of 5-3-8. Leaves were harvested at flowering stage. Only fully developed green leaves were collected, and cut to exclude the main vein.

### Sample Preparation and Phytolith Extraction

Leaf pieces and cross sections were prepared manually using razor blades. Phytoliths were isolated from mature healthy leaves using two wet extraction methods: (a) H_2_SO_4_/H_2_O_2_/HNO_3_ extraction (herein SONE), (*Protocol 2* in Corbineau et al., 2013). Leaves were cut and rinsed with 10% HCl, immersed in 70% H_2_SO_4_ solution at 70°C for 2 hours, and left overnight at room temperature. The sample was heated to 70°C, 30% H_2_O_2_ was added slowly until the supernatant became clear, and then kept heated for 3 h. The sediment was collected, rinsed with DI water thrice, and reheated to 70°C in concentrated HNO_3_ for 2 h. About 50 mg of KClO_3_ was added and the sample was kept overnight at room temperature. The sediment was collected, rinsed with DI water, washed with 0.001 M KOH solution, rinsed three times with DI water, and dried at 70°C until its weight remained constant; (b) Microwave-assisted digestion (herein MAD) using a Discover SPD-80 sample digestion system (CEM, USA). Cut leaves were oxidized by 65% HNO_3_ for 30 min at room temperature in quartz vessels, afterwards the temperature was raised linearly to 200°C over 5 min and retained for 5 min at a pressure of 200 psi. The sample was rinsed three times with DI water and dried at 70°C. Phytolith samples from both extraction methods were stored in paraffine sealed Eppendorf tubes at ambient temperature until analysis.

### Raman Microspectroscopy

Extracted phytolith samples were placed on a calcium fluoride slide without a cover slip. Raman spectra were collected from individual particles by a Jasco Raman spectrometer, using a 532-nm wavelength laser with a power of 5.6 mW for excitation, focused by a 100x objective to a spot size of ∼1 µm^2^. Spectra were obtained from 25 phytoliths of each morphology (bilobate silica cells, prickles or trichomes and long cells or plates), with 30 s acquisition time and 10 accumulations in the spectral range of 136 – 3977 cm^-1^. The spectra were calibrated using a spectrum of 4-acetamidophenol, and preprocessed with MATLAB, including background correction using asymmetric least squares method (AsLS), spectra interpolation yielding a spectral resolution of 1.8 cm^-1^, vector normalisation and selection of the spectral range of interest. PCA was performed on preprocessed spectra and on their first and second derivatives. By PCA, variations in the dataset were identified, which led to the formation of groups of similar spectra that were represented in scores plots. The loadings estimated how much each of the old coordinates, that is the wavenumbers, contributed to the PCs. Therefore, beyond differentiation and classification, PCA allowed us to highlight features in the collected dataset that are the basis for discrimination between the spectral groups, corresponding to each PC.

### Synchrotron Fourier Transform Infrared (FTIR) Microspectroscopy

Extracted phytolith samples were placed on zinc selenide slides and FTIR transmission spectra were collected from individual particles in the range from 700 to 4,000 cm^-1^ using a FTIR microscope (ThermoNicolet) at the IRIS beamline of BESSY-HZB, Berlin. The spot size from which the spectrum was acquired, was approximately 60 µm^2^ (12 × 5 µm) but was adapted to the size of each phytolith to avoid contributions by Mie scattering and maximize the signal-to-noise ratio. We collected 35 spectra of bilobate silica phytoliths and 36 long cell phytoliths. Prickle phytoliths led to strong scattering contribution to the absorbance spectra due to their morphology, and thus their spectra were excluded from the analysis. Pre­processing of the spectra included selection of the spectral range of interest, interpolation of the data, baseline correction with asymmetric least square smoothing (AsLS), and vector normalization. Extended multiplicative signal correction (EMSC) was applied to the data to correct baseline variations, noise, and scattering effects that were caused by the micron range size of the samples. The window size and polynomial order of the fitting curve for the Savitzky-Golay (SG) numerical algorithm and EMSC were optimized following a procedure previously evaluated and described (Zimmermann and Kohler, 2013). We removed nine spectra outliers during the EMSC analysis.

### Nuclear Magnetic Resonance

Solid state nuclear magnetic resonance (SSNMR) measurements were performed under magic angle spinning (MAS). Approximately 40 mg of extracted phytoliths were placed in the NMR rotor and the samples were spun at 10 kHz in all experiments. Spectra of ^29^Si Direct polarization (DP) MAS SSNMR and cross polarization (CP) MAS SSNMR were acquired at room temperature on a Bruker 11.7T Avance ІІІ spectrometer equipped with a 4-mm VTN CPMAS probe employing ^1^H decoupling at a field of 85.7 kHz. The ^1^H-^29^Si cross polarization spectra were recorded using a CP contact time of 6 ms, recycle delay of 6 s and 2048 scans. The ^29^Si direct polarization spectra were taken with a 3 μs 90° pulse followed by acquisition of 2,048 points with 8 μs dwell time a recycle delay of 60 s and 137 scans. Time domain signals (2,048 points) were zero filled to 4,096 points and multiplied by exponential decaying function (with line broadening of 100 Hz) and then Fourier transformed, phase adjusted and baseline corrected using automatic 5^th^ order polynomial function. Line deconvolutions in all ^29^Si NMR spectra shown were performed using the DMFIT program which minimizes the line shape generated by a set of simulated lines to the line shape of the convoluted spectrum (ref to https://doi.org/10.1002/mrc.984). The Q4 line in **Figure 3** was best fit by adding three more Q4 peaks aside from the main Q4 signal at −111.4 ppm (see **Table S1**). These peaks represent Q4 species with minor populations having slightly different local environments resulting from etching of the silica surface by the harsh acidic treatment. These Q4 species contribute less than 2% to the total intensity and therefore were neglected in the Q4/Q3+Q2 calculation. I.e. only the Q4 specie at −111.4 ppm was taken in calculating this ratio. The program assigns each line four parameters (position, amplitude, width, and Gaussian-to-Lorentzian ratio) which were varied until a minimum in the calculated least square function comparing the two line shapes was found. It generated a standard deviation value as a score for the goodness of fit. It also calculated the intensity percentage that each line takes, out of 100% intensity of the spectrum based on the other peak parameters. An example for the fitting parameters of the ^29^Si CP spectrum of SONE is given in the supplementary information, **Table S1**.

### Thermogravimetric Analysis

Thermogravimetric analysis (TGA) of the phytolith samples were performed with a Bargal Q500 instrument (Bargal Analy­tical Instruments Ltd, Israel) following Tishler et al. (2015). Approximately 5 mg of phytoliths were placed in a platinum crucible, equilibrated at 25°C and the weight variation recorded in the range of 30°C to 900°C under nitrogen flow of 60 ml per min, using the high-resolution sensitivity mode and a ramp of 30°C per min. Data were processed using the Universal Analysis 2000 software from TA instruments (Waters).

### Scanning Electron Microscopy - Energy Dispersive X-Ray Analysis

Leaf samples were imaged by a JCM-6000PLUS NeoScope scanning electron microscope (SEM, JEOL, Japan) at the backscattered electrons mode, under accelerating voltage of 15 kV using the low vacuum mode. Si elemental maps were obtained by energy-dispersive X-ray (EDX) with a dwell time of 2 ms, high probe current, and gain 1. Extracted phytoliths were imaged by a FEI/Philips XL-30 field emission with accelerating voltage 15 kV. Samples were mounted on a carbon tape and coated by a gold layer of 5 or 10 nm.

## Results

### Extraction Methods Affect the Structure and Chemistry of the Biosilica

**Figure 1:**
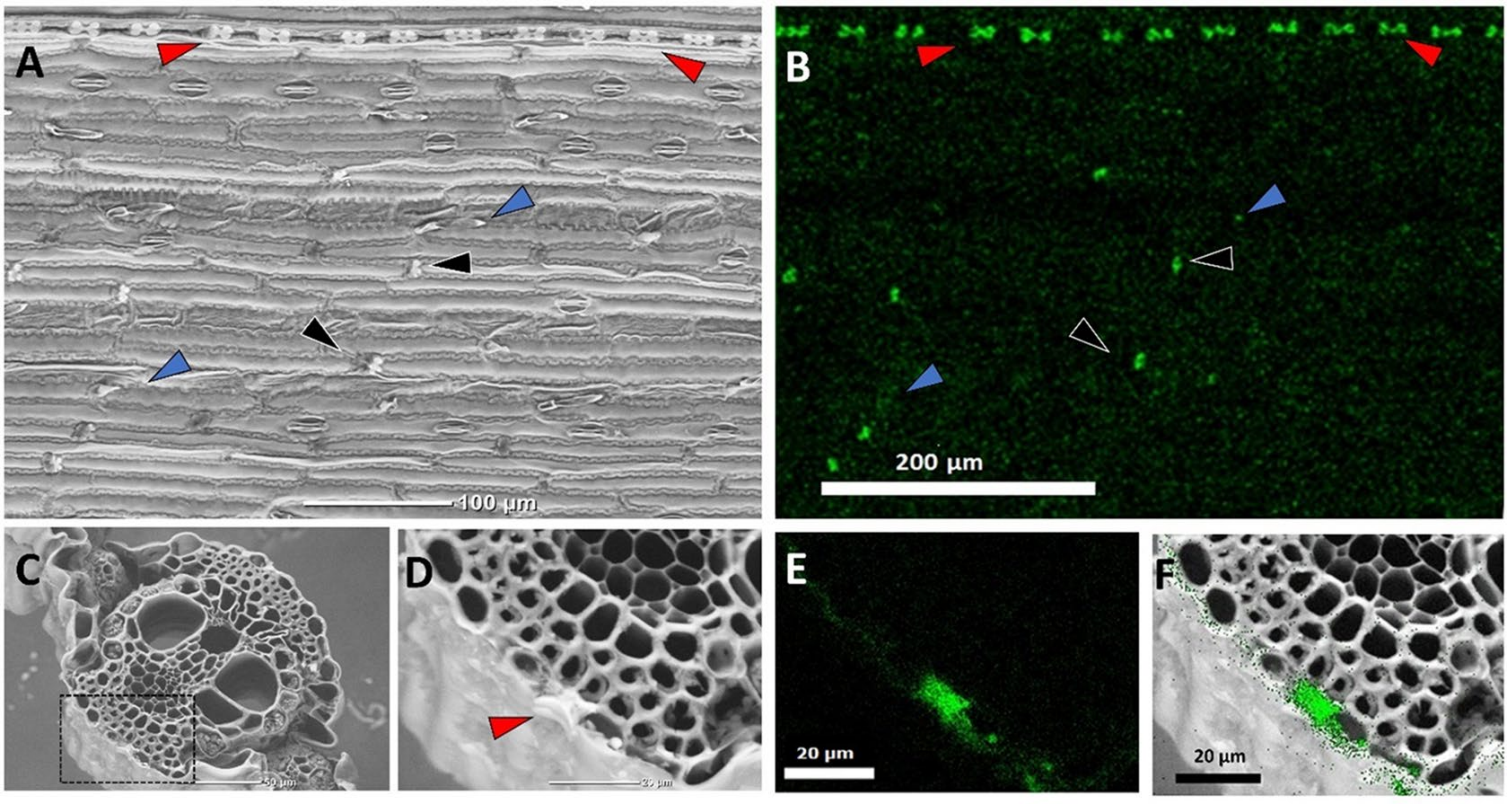
Back-scattered scanning electron micrographs (SEM) of sorghum leaves demonstrating typical silica deposition. Epidermal surface showing a row of bilobate silica cells (red arrows), cross cells (black arrows) randomly distributed between epidermal long cells, and prickles (blue arrows) **(A)**, and Si EDX map **(B)**. The white contrast in panel **(A)** matches the Si map in panel **(B)**, showing heavily silicified bilobate and cross cells, in contrast to the prickles where silica accumulates at the tips. **(C)** Leaf cross section. **(D)** Close-up of the dashed rectangle in panel **(C)** showing a bilobate cell cut transversally (arrow), and **(E)** Si EDX map of the dashed rectangle in C. **(F)** Overlay of panels **(D)** and **(E)** localizing silica to the cell walls of epidermis cells and the volume of the bilobate cell.

**Figure 2:**
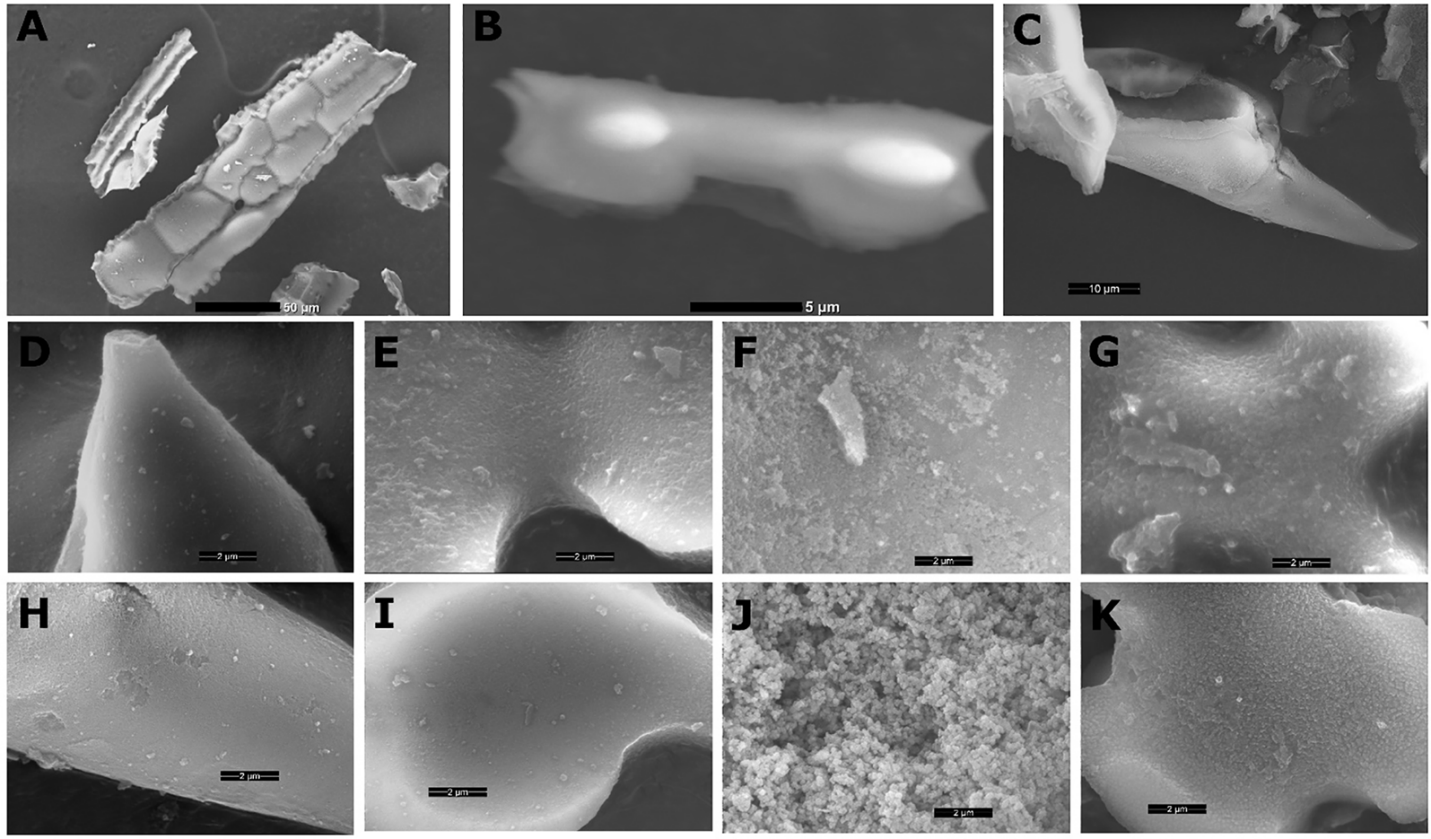
Scanning electron micrographs (SEM) of sorghum phytoliths extracted by sulphuric acid-hydrogen peroxide-nitric acid extraction (SONE) or microwaved-assisted digestion (MAD). Under low magnification (panels **A**–**C**), we did not identify differences between the extractions. **(A)** Long cells creating a silica skeleton imaged without gold coating, scale bar 50 µm. **(B)** Lateral view of uncoated bilobate silica cell showing asymmetric shape, scale bar 5 µm. **(C)** Lateral view of a prickle, scale bar 10 µm. High magnification scans of phytoliths extracted by MAD, showing tightly packed silica in a prickle **(D)**, bilobate **(E)**, long **(F)**, and cross cell **(G)**. High magnification scans of phytoliths extracted by SONE, showing a prickle **(H)**, bilobate **(I)**, long **(J)**, and cross cell **(K)**. The scale bars in panels **D**–**K** are 2 µm.

Several types of phytoliths can be found in sorghum leaf epidermis (**Figure 1**), including bilobate silica cells, silicified long cells, prickles, and cross cells, similarly to other grasses (Prychid et al., 2003). We compared plant biogenic silica isolated by two very common extraction methods: (1) sulphuric acid-hydrogen peroxide-nitric acid extraction (SONE), and (2) microwaved-assisted digestion (MAD). Both ways resulted in a similar assemblage of phytoliths, governed by long cells, bilobate silica cells and prickles (**Figures 2A–C**). Low magnification scanning electron microscopy (SEM) revealed no variation between the extraction methods. Higher magnifications of phytoliths extracted by MAD (**Figures 2D–G**) and SONE (**Figures 2H–K**) revealed spherical loosely aggregated particles in long cells only when extracted by SONE (**Figure 2J**). This finding suggested that the SONE damaged the structure of the silicon and the occluded organic matter.

#### Magic Angle Spinning - Solid State Nuclear Magnetic Resonance

**Figure 3:**
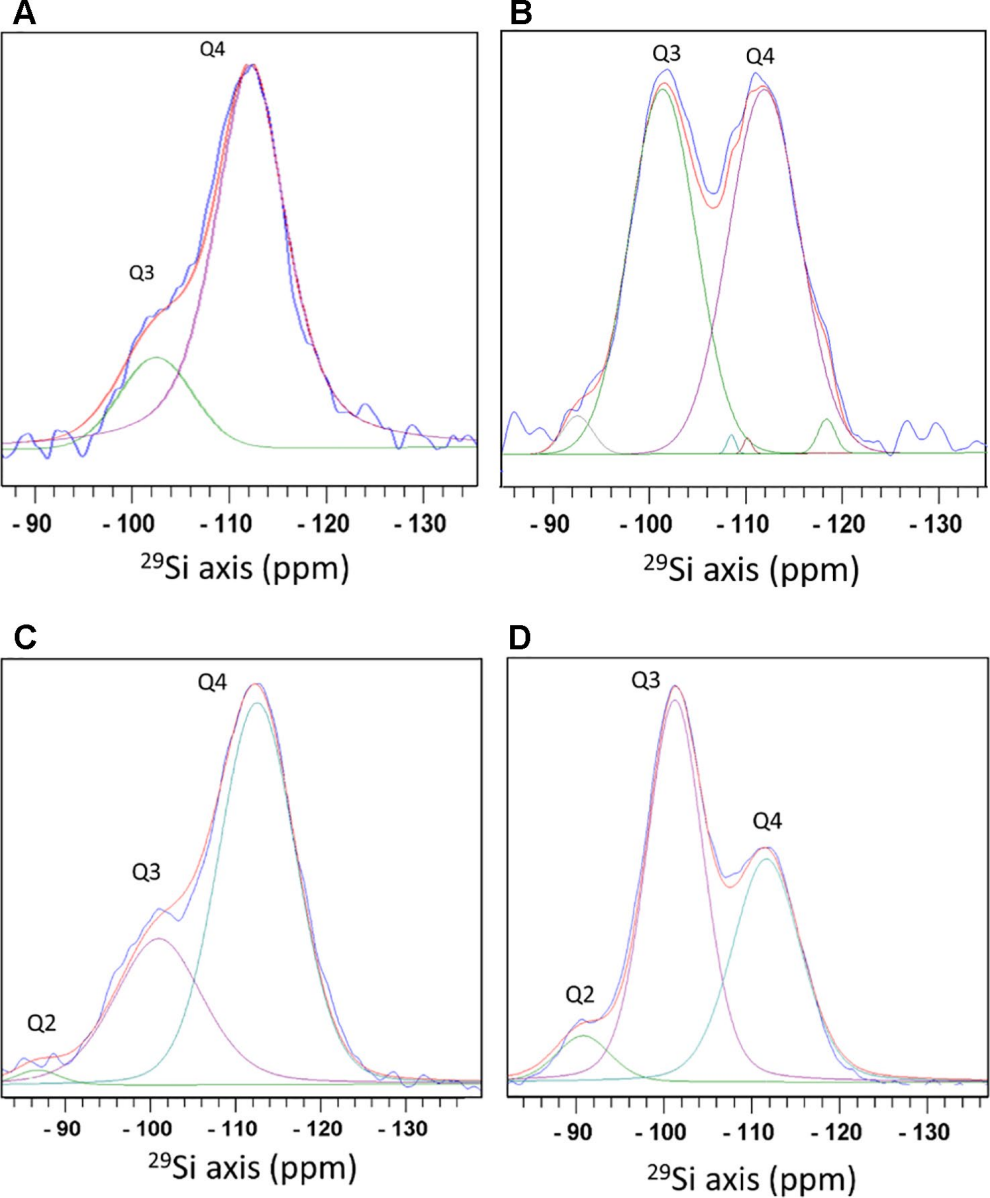
Magic angle spinning - solid state nuclear magnetic resonance (MAS-SSNMR) of ^29^Si atoms in sulphuric acid-hydrogen peroxide-nitric acid extraction (SONE) and microwaved-assisted digestion (MAD) sorghum leaf silica. Measurements of ^29^Si direct polarization spectrum (blue) **(A)**, and ^1^H-^29^Si cross polarization spectrum (blue) **(B)** of SONE silica. Optimal fit was achieved by adding minor Q4 peaks. See the fitting parameters in **Table S1**. ^29^Si direct polarization spectrum (blue) **(C)**, and ^1^H-^29^Si cross polarization spectrum (blue) **(D)** of MAD silica. Spectral decomposition into 3 lines, Q2 (green), Q3 (purple), and Q4 (cyan) is shown with the total simulated spectrum (red). Q2 corresponds to a Si atom bound to 2 hydroxyl groups, Q3 to 1 hydroxyl group, and Q4 to Si surrounded by oxygen bridging atoms with no hydroxyl groups.

Direct ^29^Si polarization (DP) spectra detected silicon atoms attached to oxygen atoms that were coordinated either to another silicon atom, or to hydrogen that formed a terminal hydroxyl. We did not identify silicon covalently bound to atoms other than oxygen. Species of O_3_-Si(OH) (termed Q3) at a chemical shift of −101.6 ppm, and O_4_-Si (Q4) at −111.3 ppm were detected in phytoliths from both extraction methods. Q2 species (O_2_-Si(OH)_2_), shifted to −91.8 ppm, were found only in the MAD (**Figures 3A, B**). The bulk (Q4) to surface (Q3+Q2) ratio was 2.9 in MAD and 4.8 in SONE samples. Selective excitation of surface Si by measuring an ^1^H-^29^Si cross polarization (CP) spectrum showed that in the SONE the Si surface species intensity ratios Q2:Q3:Q4 is 2.2:46.6:51.2. The MAD phytoliths showed the typical Si surface species intensity ratios of 6:55:39 for Q2:Q3:Q4. The siloxane to silanol ratio on the surface, calculated as Q4 to Q3+Q2, was 1.05 for SONE (**Figures 3A, B**) and was 0.64 for MAD (**Figures 3C, D**). The higher ratio in the SONE indicated a more hydrophobic surface than the surface of the MAD phytoliths.

We examined the Q2, Q3, and Q4 line intensities in the DP and CP ^29^Si spectra and compared them to values reported before for plants i.e. equisetum (Bertermann and Tacke, 2014), rice (Park et al., 2006), and diatom cell walls, called frustules (Bertermann et al., 2003; Tesson et al., 2008; La Vars et al., 2013). The bulk/surface ratio in silica from the phytoliths extracted by the MAD was similar to the ratio in native and acid extracted silica hairs of *Phalaris canariensis* (Mann et al., 1983) and extracted *C. fusiform* frustules (Bertermann et al., 2003). The bulk/surface ratio in silica from the phytoliths extracted by SONE was higher than any reported values for biosilica.

The relative intensities of the Qn lines in the CP spectrum are dependent on parameters of the experiment and sample properties. For example, the CP spectra measured on phytolith silica using a CP contact time of 6 ms and a recycle delay of 6 s are roughly comparable to diatom CP spectra measured with a CP contact time of 5 ms and a recycle delay of 4 s (Bertermann et al., 2003). The Q4/(Q3+Q2) ratio in CP of phytolith silica is, therefore, crudely compared to the ratios in other reported biosilica samples. This ratio in silica extracted by the MAD is similar to the ratio in *E. giganteum* (Bertermann and Tacke, 2014) and dried extracted frustules of several diatoms such as *C. fusiformis* (Bertermann et al., 2003) and *T. pseudonana* (Tesson et al., 2008). In silica extracted by SONE, this ratio is similar to the value reported for dried *C. muelleri* diatom grown in high salt concentrations (La Vars et al., 2013). The seminal early report by Perry does not contain details on cross polarization times (Perry, 1985).

#### Thermogravimetric Analysis

**Figure 4:**
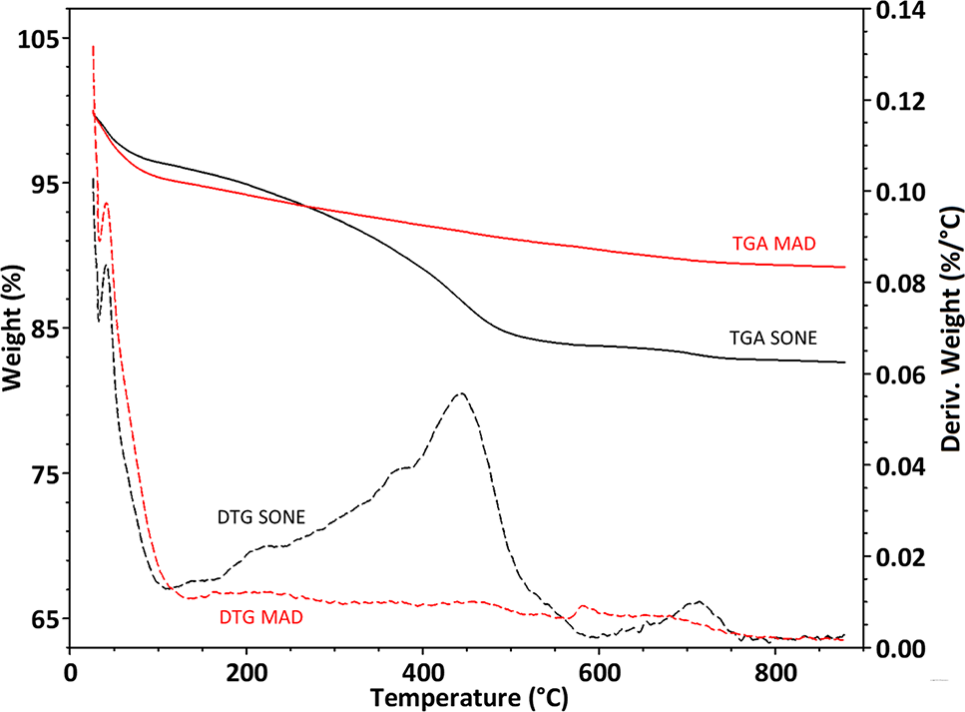
Thermogravimetric analyses of extracted phytoliths. Curves of percent weight loss (full line, left Y-axis) and derivative of weight loss by temperature (dashed line, right Y-axis) of the microwaved-assisted digestion (MAD) (red) and sulphuric acid-hydrogen peroxide-nitric acid extraction (SONE) (black) samples.

After pyrolysis, the SONE sample lost about 17% of its weight, while the MAD sample lost only about 12% (**Figure 4**). The weight loss is assumed to be composed mainly of bound water (up to 150°C) and organic matter (150°C – 800°C). We identified a peak in the weight loss rate at 120°C (**Figure 4**), associated to bound water, and representing 4.9% of the weight of MAD and 3.8% weight of the SONE sample. The TGA is consistent with our NMR analysis, showing a more hydrophilic character of the silica extracted by MAD. Differential thermal gravimetric (DTG) broad peaks at 250°C, 380°C, 450°C, and 700°C appear only in the SONE sample (**Figure 4**). The lack of peaks in the DTG of the MAD phytoliths indicates that much less organic matter remained after this extraction. The continuous weight loss between 150°C to 800°C in both extraction methods can be attributed to removal of chemically bound water (OH in the surface of silica powders), (Mueller et al., 2003), changing the surface chemistry from silanol to siloxane groups.

### Microspectroscopic Characterization of Individual Phytoliths

#### Raman Analysis

**Figure 5:**
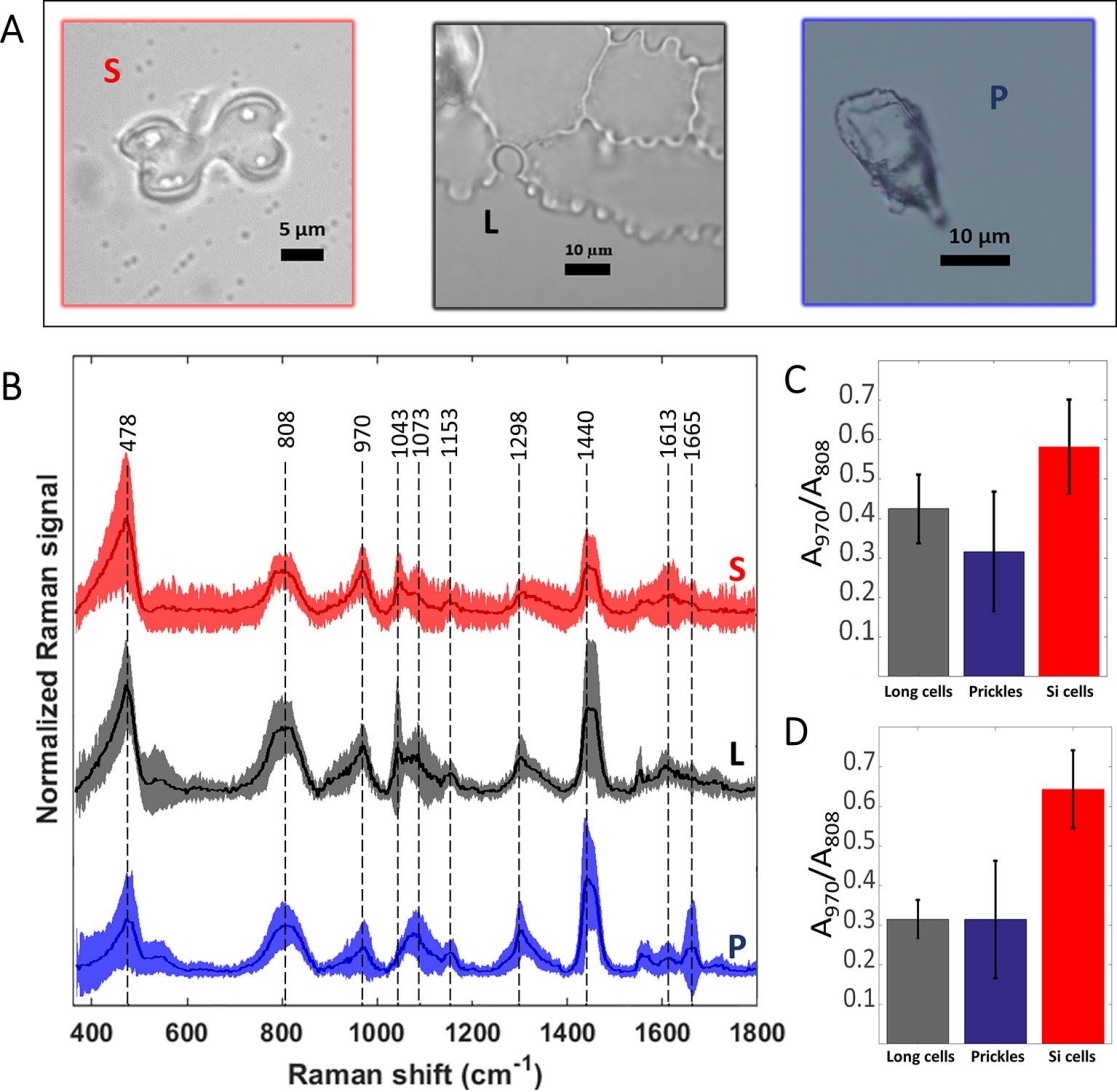
Preprocessed mean Raman spectra of the most abundant sorghum leaf phytoliths and intensity ratio of the Si-OH to Si-O-Si Raman bands. Bilobate silica cells (S, red), long cells or plates (L, black) and prickles (P, blue) are shown in bright-field micrographs **(A)**. Mean spectra ± standard deviation are plotted in the same respective colour and denoted with the same abbreviations **(B)**. Averages of 25 spectra of phytoliths of each type extracted by microwaved-assisted digestion (MAD) are shown. The area of the peak at 970 cm^-1^, assigned to Si-OH surface groups, was normalized to the area of the 808 cm^-1^ band, assigned to Si-O-Si stretching. Ratios of band areas calculated in spectra of bilobate cells were significantly higher (p < 0.05) than both prickles and long cells under MAD **(C)** and sulphuric acid-hydrogen peroxide-nitric acid extraction (SONE) **(D)** methods.

We measured Raman spectra of prickle, long, and bilobate phytolith cells (**Figures 5A, B**). Due to the noncrystalline and nonuniform molecular structure of the silica, the Raman bands were broad. The signal extending from 400 to 500 cm^-1^ with a maximum at 478 cm^-1^ (Si-O-Si bending modes) was assigned to five-, six-, and seven-membered SiO ring (Sharma et al., 1981). Other characteristic Raman silica bands appeared at 808 cm^-1^ (Si-O-Si symmetric stretching), 970 cm^-1^ (Si-OH stretching mode of nonbridging oxygen atoms) and 1,070 cm^-1^ (Si-O-Si asymmetric bond stretching), (Bertoluzza et al., 1982). To estimate the hydroxyl density in the different phytoliths we calculated the intensity ratio of the band at 970 cm^-1^ to that at 808 cm^-1^ after AsLS baseline correction (**Figures 5C, D**). The latter band was used for normalization because it is a lattice band characteristic to the silica network and remains unchanged in different silicas (Humbert, 1995). The ratios calculated for bilobate cells were significantly higher than those ratios calculated for both prickles and long cells under MAD and SONE (p < 0.05, T-test). No significant differences were detected between long cells and prickles. Our results suggest a larger surface to volume ratio and a lower degree of condensation of the silica in the bilobate cells in comparison to that in prickles and long cells.

All other bands in the spectrum were attributed to organic matter occluded within the silica: C-C twisting and rocking at 1,153 cm^-1^, CH_2_ deformation in alkane long chains at 1,298 cm^1^, and CH_2_ deformation vibrations in n-alkanes at 1,440 cm^-1^ (Parker, 1983). A small band at 1,613 cm^-1^ was attributed to C = C stretching or aryl stretching vibrations was also identified. We associated it with the presence of modified lignin (Ram et al., 2003). Prickle cells presented two unique features: a band at 1,665 cm^-1^ that was assigned to the C = C stretching, C = O stretching, and amide I vibrations, and the absence of a band at 1,043 cm^-1^, which was assigned to ring vibrations of substituted benzenes and C-C stretches in n-alkanes (Parker, 1983), (**Figure 5B**). Raman spectra of prickles were the only place we could identify contributions that are typical to proteins, in peaks associated to amide I (1,600–1,690 cm^-1^) and amide II (1,480–1,580 cm^-1^), (Tuma, 2005).

**Figure 6:**
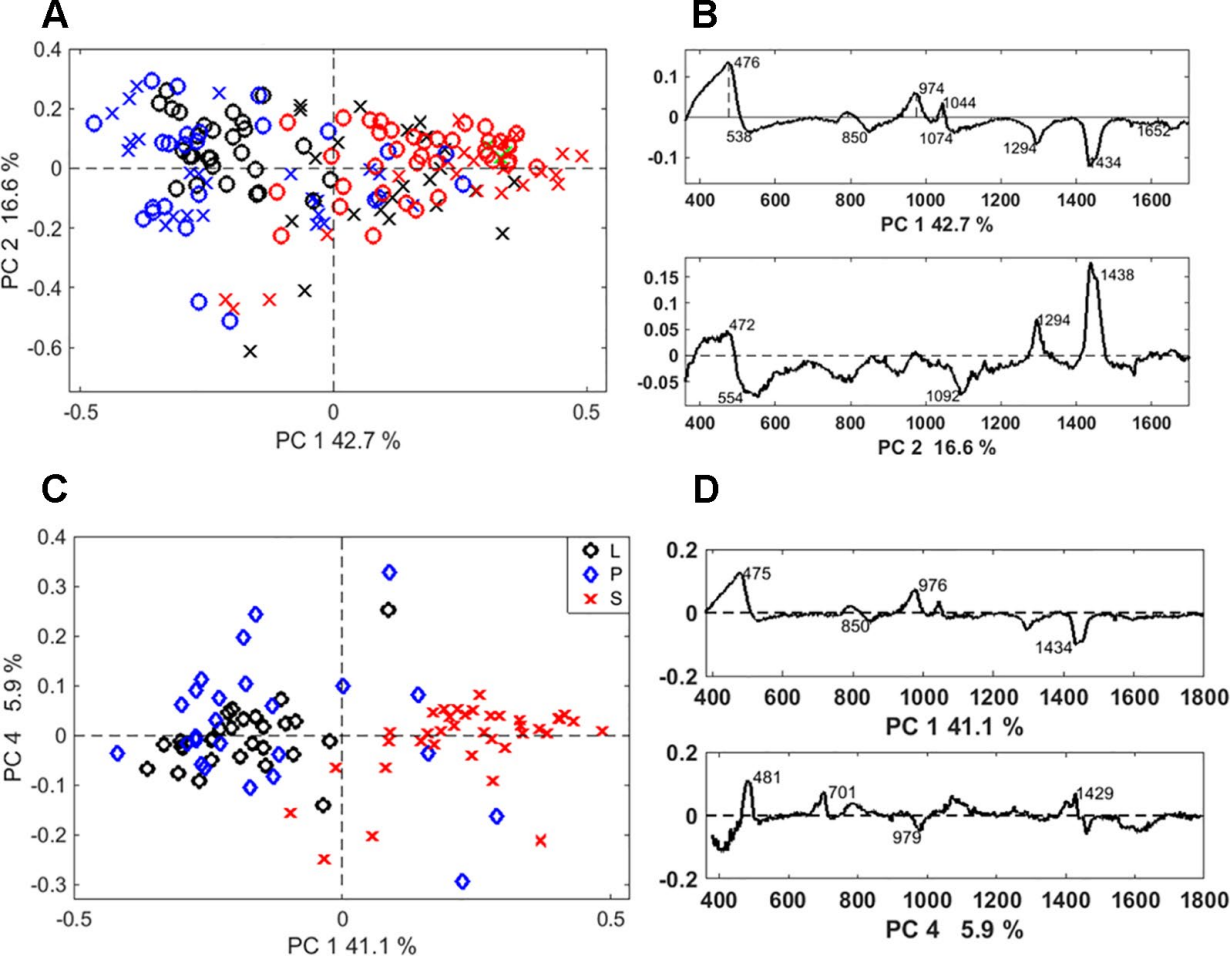
Discrimination of Raman spectra of individual phytoliths. **(A)** Principal component analysis (PCA) scores plot of the phytolith Raman spectra showing discrimination between phytolith types: prickles (blue), long cells (black), and bilobate silicified cells (red), extracted by microwaved-assisted digestion (MAD) (X) or sulphuric acid-hydrogen peroxide-nitric acid extraction (SONE) (O). **(B)** Loading spectra of PC1 and PC2, indicating bands responsible for the separation. **(C)** PCA scores plot showing the discrimination between phytolith types extracted by SONE (long cells (L, black), prickles (P, blue), and bilobate cells (S, red)). **(D)** Corresponding loading spectra of PC1 and PC4.

Discrimination between the two extraction methods was achieved by PCA of the Raman spectra. The separation was particularly clear when the PCA was applied to the spectra of long cells (**Figure S1A**). In this case, the loading spectra that indicate the source of the variation, revealed differences in the silica structure and the amount of occluded organic matter (**Figure S1B**). Based on PCA, a separation between different phytolith types was possible regardless of the extraction method (**Figure 6A**). A clear separation between the bilobate and long cells was achieved when we analysed only the SONE phytoliths spectra (**Figure 6C**). The source of separation was studied based on the PCA loadings (**Figures 6B, D**). In both the full dataset as well as the SONE dataset the highest variation, which is represented by PC1, is explained by an increase in the bands at 475, 808 and 970 cm^-1^ and a decrease in the band at 1,435 cm^-1^. In the scores plot, long cells and prickles appeared at negative values of PC1, indicating a higher contribution of the 1,435 cm^1^ CH_2_ deformation band, associated with lipids (Parker, 1983). The other bands that contribute to the variance represented by PC1 corresponded to vibrational modes of Si-O-Si and Si-OH.

PCA was also applied to the derivatives of the Raman spectra (**Figure S2**). We found high variation within the group of the bilobate cells in comparison to the long cells and prickles that formed a compact distribution in the scores plot. The discrimination was based on differences in the shape of bands between 440 and 500 cm^-1^, indicating differences in the structure of the silica.

#### Synchrotron Infrared Microspectroscopy

**Figure 7:**
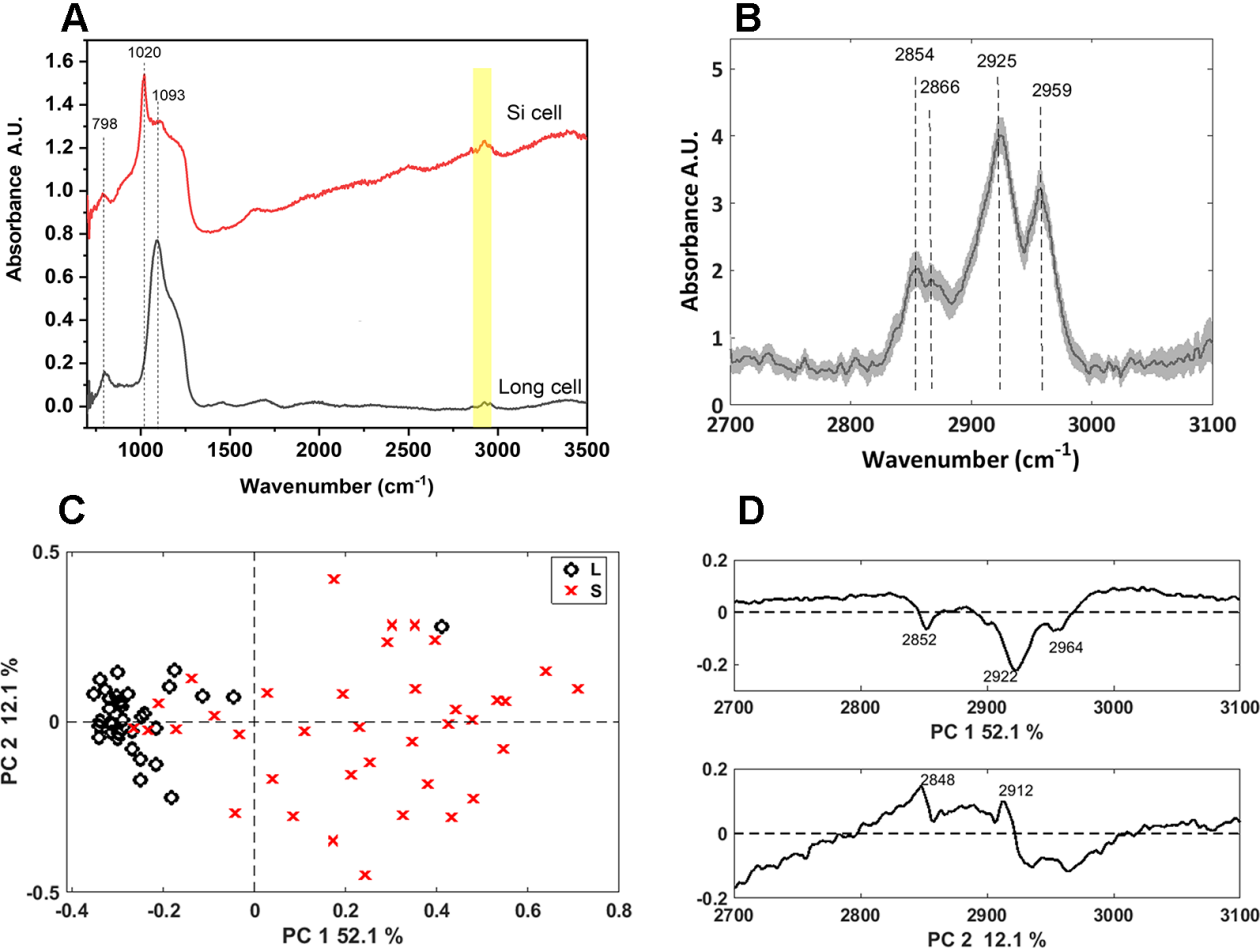
Synchrotron Fourier transform infrared (FTIR) spectral analysis of long and bilobate cells extracted by sulphuric acid-hydrogen peroxide-nitric acid extraction (SONE). **(A)** Representative spectra of long and bilobate cells. Yellow shade at 2,700–3,000 cm^1^ showing bands typical to hydrocarbons. **(B)** Average FTIR spectra ± standard error of long cell phytoliths in the range 2,700 to 3,100 cm^-1^. **(C)** Scores plot of a PCA at the spectral region 2700–3000 cm^1^, attributed to the organic matter occluded in long (L) and silica (S) cells, discriminating between the two phytolith types. **(D)** Loadings of the principal component analysis (PCA) correlate the discrimination with the terminal -CH_3_ and CH_2_ groups absorption bands.

We further characterized the long and bilobate cells extracted by SONE by FTIR microspectroscopy (**Figure 7**). The main spectral features were attributed to the silica: the band at 800 cm^-1^ are assigned to the deformation of Si-O-Si bonds bridging between two adjacent tetrahedral (Kirk, 1988), and the bands at 1,000–1,250 cm^1^ are assigned to Si-O asymmetric stretching modes. The latter band has a maximum at 1,093 cm^-1^ in the phytoliths of long cells, and at 1,020 cm^-1^ in bilobate cells (**Figure 7A**). This variation indicates differences in the silica structure between the phytolith types. PCA analysis supported this observation (**Figure S3**), resulting in clear separation of the two cell types. Infrared bands in the 2,700–3,100 cm^-1^ region suggested that a considerable amount of organic matter remained linked to the extracted silica. The spectra of the long cells display bands at 2,854, 2,866, 2,925, and 2,959 cm^-1^ (**Figure 7B**), which are attributed to C-H stretching in -CH_3_ and CH_2_ groups (Silverstein et al., 2005). These bands are expected in biological materials due to the presence of terminal -CH_3_ and of CH_2_ groups in cellular components like proteins, carbohydrates, and lipids. PCA of the spectra in the 2,700–3,100 cm^-1^ range separated the long cells and the bilobate cells along PC1 (**Figure 7C**). The loading of PC1 represents absorption bands of organic matter (**Figure 7D**). However, these spectral features are represented in negative values. Therefore, the negative values for PC1 coefficients, at which the long cell phytoliths spectra are found (**Figure 7C**), lead to the conclusion that more organic material must be occluded in the long cell phytoliths as compared to the bilobate cells.

## Discussion

In this work, we aimed to discriminate between phytolith types extracted from the same sorghum leaf. Our hypothesis was that the phytoliths that are produced by varied cell types will vary in their occluded organic matter. We also assumed that the harsh extraction conditions alter and mask genuine variations between phytolith types (Alexandre et al., 2015). Our NMR and Raman data indicated that the extraction changes the silica structure. The number of silanol groups on the silica surface was lower in phytoliths extracted by SONE in comparison with the MAD (**Figure 3** and **Figure S1**), making SONE silica less polar and more hydrophobic. TGA supported this by showing lower percentage of water molecules released below 150°C in the SONE sample (**Figure 4**). Our TGA measurements further showed that the SONE was less aggressive than the MAD, which left hardly any organic matter in the phytoliths. SEM indicated that the long cells behaved differently under the two extraction methods, in accordance with the Raman PCA that could discriminate between the extractions based on long cells spectra. Our results clearly show that long cells react differently to the extractions. More research is needed to elucidate the native state of the silica and its occluded organic matter as synthesized in the plant.

Using single particle spectroscopy, we could show that under the same extraction, the silica structure is different between phytolith types. In general, IR vibrational spectra of microscale particles are masked by Mie scattering that depends on the particles’ shapes. Even so, the spectra of bilobate phytoliths show a prominent shift to lower energies in the Si-O asymmetric stretching vibration as compared to long cells. This difference indicates variation in the atomic organization of the mineral. Our results thus conform with the hypothesis that silica organization is under biological control, as was suggested by Perry et al, showing that variation in the mineral nanostructure in correlation to cell developmental stages (Perry and Mann, 1989) correlates to the silanol groups exposed on the silica surface (Perry et al., 1990). In agreement with FTIR, the PCA of the Raman spectra resulted in the formation of two groups: one includes spectra of bilobate silica cells and another of prickles and long cells. The Raman spectra indicated a larger ratio of surface to bulk Si atoms in the bilobate cells in comparison to prickles and long cells (**Figures 5C, D**). These differences may arise from higher number of silica nucleation sites in bilobate cells in comparison to long cells and prickles. Biogenic moieties that integrate to the bulk mineral or attach to its surface may also alter the mineral structure. The variation in the mineral structure was persistent within a phytolith type, suggesting that within the same cell type, similar plant factors interact with the mineral, and these materials may differ between cell types—specifically between bilobate and long cells.

From our results, it is not possible to determine the hydroxylation degree of the native biosilica before its extraction. However, the variations between phytolith types extracted similarly indicate either an initially distinct variation in hydroxylation or structure of the silica of different phytolith types. Regardless of the actual origin of the variation in hydroxylation degree, it most probably indicates that there is more than one pathway of silica deposition in sorghum leaves.

The SONE allowed us to analyse organic matter that was intimately associated with the silica (**Figure 4** and **Figure S1**). Our results indicated that the Si atoms are coordinated to oxygen, similarly to silica gel and opal, in agreement with analyses of *in planta* silica (Yoshida et al., 1959; Casey et al., 2004) and *in vitro* precipitation with lignin (Cabrera et al., 2016; Soukup et al., 2019). We cannot exclude the existence of Si-O-C bonds as detected by X-ray photoelectron spectroscopy in cell walls extracted from rice cell suspension (He et al., 2015). These bonds may be below the detection limit because obviously they are not abundant, and their vibrations are expected at very similar energies to Si-O-Si vibrations. In addition, they may wash out or decompose during extraction.

Si in cell walls of *Equisetum arvense* is associated with cell wall polymers, including polysaccharides, proteins, and phenolic acids, suggesting that silica may form in a range of chemical conditions independent of a charged matrix (Currie and Perry, 2009). Raman and Infrared bands associated to lipids were more intense in the spectra of long cell and prickle phytoliths, suggesting that the cuticle incorporated into the mineral (**Figure 7**). This is in agreement with the existence of a cuticle-silica double layer, observed first in the epidermis of rice by Yoshida et al. (1962). Cell wall polymers (possibly polysaccharides) are involved in the deposition of silica in hairs and epidermis, similarly to hairs and outer epidermis cells in lemmas of the grass *Phalaris canariensis* (Hodson et al., 1984; Perry et al., 1987). In comparison to long cells and hairs, we found that the mineral in bilobate cells contained lower fraction of organic residues. In sorghum bilobate cells silica deposits between the cell membrane and wall, constricting the protoplast and creating a secondary wall made of silica (Kumar and Elbaum, 2018). Thus, the bilobate silica deposition pathway excludes cuticle materials and includes only small amounts of cell wall polymers in the mineral.

Acidic proteins and glycoproteins are found in association with mineral phases as components of the organic matrix encapsulated in phytoliths (Harrison, 1996; Elbaum et al., 2009). Specifically in bilobate, protein residues were identified embedded in their silica (Alexandre et al., 2015). A protein (Siliplant1) was identified inside sorghum bilobate cells that is active in *in planta* silica deposition (Kumar et al., 2019). Nonetheless, our results did not provide direct evidence of amino acids in bilobate cells, possibly because they degraded during the phytolith extraction. We suggest that other organic compounds such lipids and carbohydrates are much more abundant than proteins in the extracted sorghum phytoliths. The presence of more organic matter entangled within the silica of long cells and prickles in comparison to bilobate phytoliths may be explained by a slow co-deposition of silica and other cell wall components like lignin, cutin, hemicelluloses, and cellulose (Perry et al., 1987; Fry et al., 2008; Law and Exley, 2011; Soukup et al., 2017; Kulich et al., 2018). The observed differences in hydroxylation and amount of occluded organic matter between phytolith types are also expected to have an effect on the dissolution rate of phytoliths (Nguyen et al., 2019).

## Conclusions

Due to the strong influence of the method used to extract the phytoliths on the silica structure and occluded organic matter, it is important to study plant silicification *in situ* in the native tissues. Differences between phytolith types extracted similarly from the same leaf suggest that the mineral deposits through a cell type-dependent pathway. Two mechanisms are suggested by our data: one involves the mineral impregnation of a cuticle-cellulose matrix (in long cells and prickles) and another suggests a low fraction of organic matrix (in bilobate silica cells) on which silica deposits.

## Data Availability Statement

The raw data supporting the conclusions of this manuscript will be made available by the authors, without undue reservation, to any qualified researcher.

## Author Contributions

VZ, JK, and RE planned the research, designed the study, and wrote the manuscript. VZ collected the TGA data, Raman, and IR spectra, and SEM images; NF and GG performed the NMR experiments. VZ and SD analyzed the data. All authors commented, added, and revised the manuscript and approved for publication.

## Funding

This work was funded in part by the Excellence Initiative of the German Research Foundation (DFG) GSC 1013 (SALSA) and the Israel Ministry of Agriculture grant 12-01-0031. We thank BESSY-HZB for the allocation of synchrotron radiation beam time.

## Conflict of Interest

The authors declare that the research was conducted in the absence of any commercial or financial relationships that could be construed as a potential conflict of interest.

The handling editor is currently organizing a Research Topic with one of the authors RE, and confirms the absence of any other collaboration.
