# Supplementary material for: Spectroscopic Discrimination of Sorghum Silica Phytoliths
Source: Front Plant Sci. 2019 Dec 11;10:1571. doi: 10.3389/fpls.2019.01571 (PMC6917640; doi:10.3389/fpls.2019.01571)
Supplement: Supplementary file 1 [file DataSheet_1.docx]

Supplementary Material

**Table S1 │ Fitting parameters produced by DMFIT for the ^29^Si CP spectrum of SONE.** Gaussian-to-Lorentzian ratios were 1 in all Si forms. The Q4 line in Figure 3B was best fit by adding three more Q4 peaks aside from the main Q4 signal at -111.4 ppm. These peaks represent Q4 species with minor populations having slightly different local environments, resulting from etching of the silica surface by the harsh acidic treatment.

|  | **Q2** | **Q3** | **Q4** | **Q4** | **Q4** | **Q4** |
| --- | --- | --- | --- | --- | --- | --- |
| **Amplitude (arbitrary units)** | 12.5 | 117.5 | 117.3 | 6.03 | 5.04 | 11 |
| **Position (ppm)** | -93.16 | -101.5 | -111.4 | -108.2 | -109.8 | -117.5 |
| **Line width (ppm)** | 3.5 | 7.7 | 8.2 | 0.98 | 0.96 | 2 |
| **% Integrated lntensity** | 2.3 | 46.6 | 49.5 | 0.3 | 0.3 | 1.1 |

*


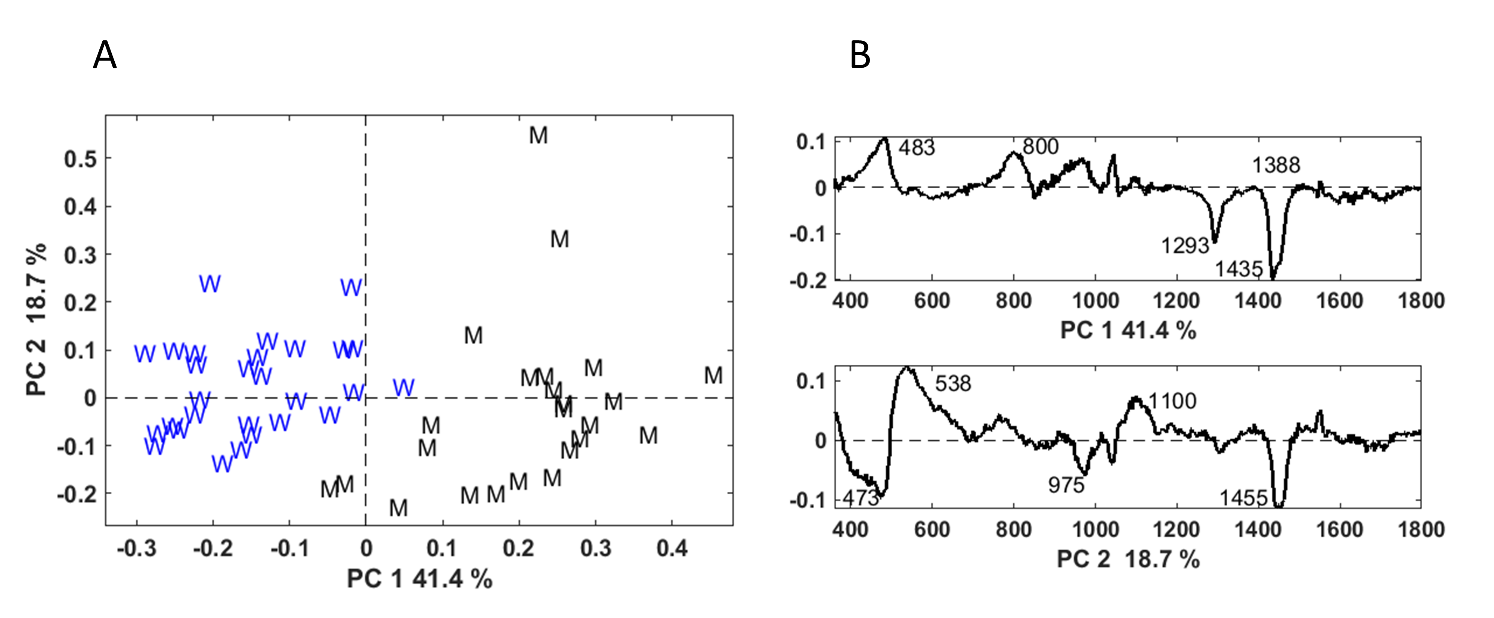
 **Figure S1│** **Discrimination between extraction methods by Raman microspectroscopy.** (**A**) PCA scores plot of long cell phytoliths (plate fragments). W in blue represents long cells extracted by SONE and M in black represents long cells extracted by MAD. **(B)** PCA loadings showing the contributions of original variables to PC 1 and PC 2 in panel A. The contribution to the loadings of the first principal component indicate that the separation was based on the organic matter present in the silica and the amount of silanol groups on its surface (bands at 483, 800 and 970 cm^-1^). The band around 800 cm^­1^ was assigned to the Si-O-Si symmetric stretching mode, and at 975 cm^-1^ to silicon-oxygen stretching modes of Si-OH groups (Bertoluzza et al., 1982).


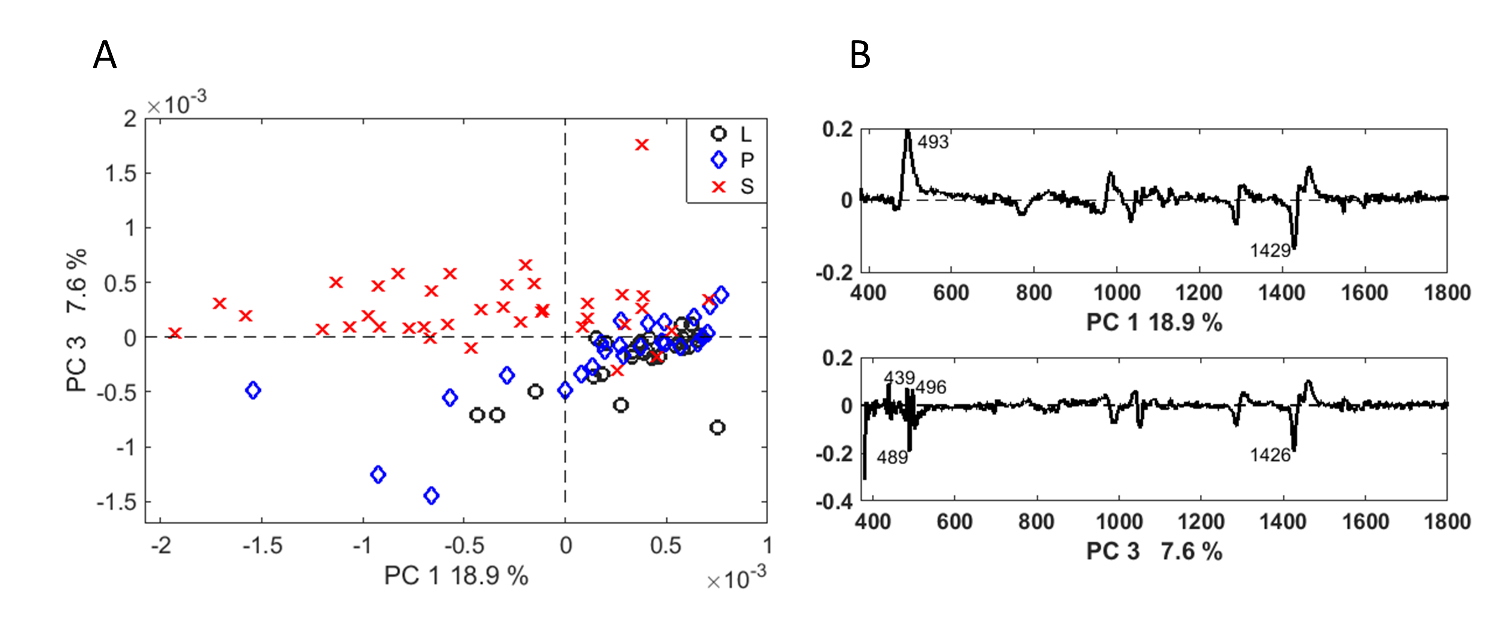


**Figure S2│ Discrimination between phytolith types based on first derivative Raman spectra of SONE** **phytoliths.** (**A**) PCA scores plot of long cells (L, black); prickles (P, blue); and bilobate cells (S, red). The long cells are more compactly grouped whereas the bilobate cells showed higher variation. (**B**) Loadings of the PCA. The variance explained by the principal components is smaller than in the analysis of the Raman intensity spectra **(Figure 7)**. This is because the spectral derivation removes structural information that is contained in the spectra baseline. According to the loadings of the PCA, the discrimination was based on the differences in the shape of the band between 440 and 500 cm^-1^.

**
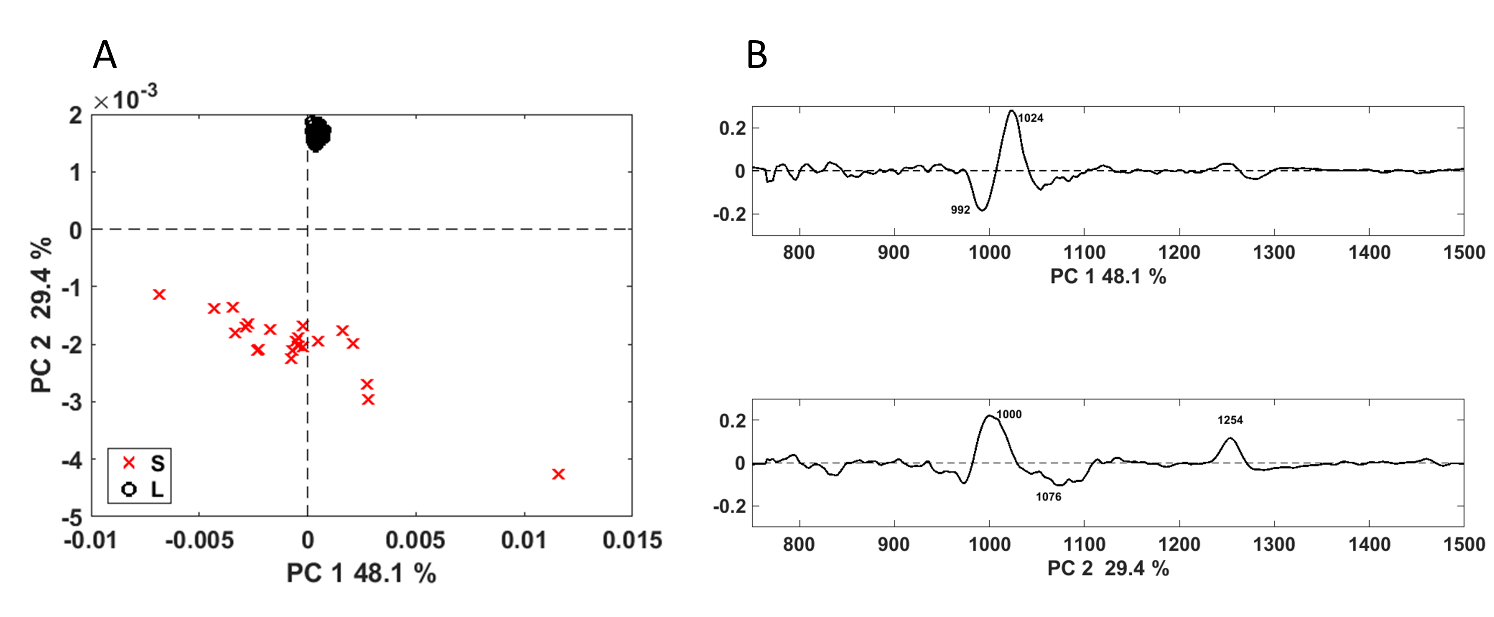
**

**Figure S3│ Discrimination between phytolith types based on Infrared spectra second derivatives.** Synchrotron Fourier transform infrared spectra were collected from long (L) and bilobate (S) cells extracted by SONE. **(A)** PCA scores plot of the spectral second derivative in the range of 700-1500 cm^­1^ showing strong separation between the cell types. The silica cells exhibit high variation in the scopes of both PC1 and PC2 whereas the long cells seemed to be more homogeneous. **(B)** Loadings of the PCA indicate differences between the phytolith types in the band around 1000 cm^­1^, related to the silica structure, in agreement with the Raman analysis (**Figure S2**).
